# Effects of wetted inner clothing on thermal strain in young and older males while wearing ventilation garments

**DOI:** 10.3389/fphys.2023.1122504

**Published:** 2023-02-22

**Authors:** Ken Tokizawa

**Affiliations:** National Institute of Occupational Safety and Health, Tokyo, Japan

**Keywords:** core temperature, sweat loss, aging, cooling, occupational heat strain

## Abstract

The present study examined the effect of wearing a water-soaked inner t-shirt with a ventilation garment on thermal and cardiovascular strain in eight young (26 ± 4 years) and eight older (67 ± 3 years) men undertaking moderate-intensity work (metabolic rate: 200–230 W m^−2^) in a hot environment (37°C, 50% RH, 2.8 kPa). While intermittent walking in hot conditions for 60 min, as a control (CON), the subject wore a dry inner t-shirt (long-sleeved) without fanning of a ventilation jacket (single-layered cotton, 0.21 clo). On separate days, under a fanned ventilation jacket, the subject wore a dry inner t-shirt (DRY) or an inner t-shirt soaked with 350 mL of tap water (WET). In the young group, increases in rectal temperature from pre-exercise baseline in the WET trial (0.7°C ± 0.2°C) were lower than in the CON (1.3°C ± 0.3°C) and DRY (1.1°C ± 0.2°C) (both *p* < 0.05) trials during exercise in hot conditions. In the older group, the increases were also attenuated in WET (0.7°C ± 0.4°C) compared with CON (1.3°C ± 0.4°C) and DRY (1.1°C ± 0.4°C) (both *p* < 0.05) without differences between age groups. Heart rate and whole-body sweat loss were lowest in the WET, followed by DRY, and then CON conditions in both groups (all *p* < 0.05). These findings demonstrate that wearing a water-soaked inner t-shirt while using a ventilation garment is an effective and practical cooling strategy to mitigate thermal and cardiovascular strains in young and older individuals during moderate-intensity work in hot conditions.

## Introduction

Climate change and global warming cause increased human exposure to more prolonged and intense heat, which hampers labor productivity in physically demanding work (Flouris et al., 2018). At the same time, labor force demographics are rapidly changing and the unprecedented aging of populations and workforce in most developed and many developing countries has significant implications for employees, human resource management, organizations, and societies (Hertel and Zacher, 2018). Older individuals are vulnerable to heat stress, which is associated with an alteration in thermoregulatory function both in hot environmental conditions and during exercise (Kenney et al., 2014). Reduced sweating response and cutaneous vasodilation impair heat loss (Inoue and Shibasaki, 1996; Kenney and Munce, 2003), which induces hyperthermia, higher levels of cardiovascular strain (Kenney et al., 2014), and increases the risk of heat-related illness during exposure to a hot environment and exercise (Kenney and Munce, 2003). To protect an aging workforce, especially in physically demanding jobs in hot conditions, countermeasures for inhibiting an elevation of core temperature (T_core_) by body cooling strategies should be reconsidered or developed, because cooling effects (e.g., fanning) might be less effective in older adults than in young ones due to the reduced heat dissipation (Gagnon et al., 2016; Gagnon et al., 2017).

Personal cooling garments have been found to attenuate thermal and cardiovascular strains during working, such as ambient or cold air ventilation, liquid cooling, and phase-change material garments (Yazdi and Sheikhzadeh, 2014; Chan et al., 2015). These garments have inevitable ergonomic problems such as the additional weight and layers, and restriction of body movement, but an ambient-air ventilation garment can be practical. The attached fans and mobile battery are light, and the garments do not bother the wearer during work activities. However, a field study showed that the efficacy of ventilation garments was limited to reducing skin temperature (T_skin_), then T_core_, heart rate (HR), and labor productivity were not affected by their use in hot conditions (Ioannou et al., 2021).

In a laboratory, Shapiro et al. (1982) showed that ventilation garments attenuated increases in T_core_ and HR during exercise at 35°C (75% RH) while wearing a protective semipermeable overgarment. Since then, ventilation units have been improved and evaluated in human subjects (Muza et al., 1988; Chen et al., 1997; Chinevere et al., 2008; Hadid et al., 2008; Barwood et al., 2009) and by modeling using thermal manikins (Xu and Gonzalez, 2011; Zhao et al., 2013; del Ferraro et al., 2021; Yang et al., 2022); the efficacy of using ventilation garments might be potentially augmented. Although evaporation depends on the wearer’s sweat, the volume of sweat varies between individuals (Bain et al., 2011) and with age (Shoenfeld et al., 1978; Inoue et al., 1991). To this end, soaking inner shirts with water can supplement sweat production while using ventilation garments, which might be an ideal strategy for older individuals.

Some studies have demonstrated that supplemental skin wetting is less effective for mitigating thermal and cardiovascular strains during climatic heat waves. Song et al. (2019) reported that soaking a t-shirt and short pants inhibited increases in the T_core_ by ∼0.2°C while resting at 43°C (57% RH) for 90 min. Morris et al. (2019) showed that self-dousing with water to the skin attenuated increases in HR by ∼5 beats/min and sweat losses resting at 40°C (50% RH) and 47°C (10% RH) for 120 min without affecting T_core_. Cramer et al. (2020) reported that soaking a t-shirt attenuated the increases in T_core_ by ∼0.2°C, HR by ∼5 beats/min, and sweat losses in resting at 42°C (34% RH) for 120 min. However, they also showed that electric fan use with a water-soaked t-shirt abolished these mitigating effects. Because the T_a_ exceeded the T_skin_, it seems that fanning in such conditions produced convective heat gain and offset any improvement in evaporative heat loss (Cramer et al., 2020). A recent study demonstrated that the beneficial thresholds for electric fan use were for temperatures below 37°C (Morris et al., 2021). Although previous studies targeted daily living in a house designed to resist heat waves (Morris et al., 2019; Song et al., 2019; Cramer et al., 2020), workers rarely experience extreme heat or avoid working in such conditions. Daytime hot weather conditions in most countries worldwide were under the threshold in recent years with a broad range of humidity (5%–80% RH) (Morris et al., 2021). Thus, the threshold of T_a_ of 37°C and the middle of the RH range of 50% were adopted in the present study for assessing the effects of combining a wet-inner t-shirt with ventilation garments.

The aim of this study was to evaluate whether a water-soaked t-shirt while wearing ventilation garments would attenuate exercise-induced thermal and cardiovascular strains in young and older men at moderate-intensity work (200–230 W m^−2^, the average metabolic rate of physically demanding occupations) in hot and moderately humid conditions. It was hypothesized that 1) wearing a water-soaked t-shirt in combination with a ventilation garment would attenuate thermal and cardiovascular strains compared with a dry t-shirt/ventilation garment combination in both age groups; and 2) mitigation of thermal and cardiovascular strains would be greater in the older than in the young group.

## Methods

### Participants

Sixteen healthy men were recruited for this study from two age ranges: eight young (age 26 ± 4 years, range 21–30) and eight older (age 67 ± 3 years, range 62–70). There were no differences between groups for height (young, 171 ± 5 vs. older, 166 ± 5 cm; *p* = 0.14), body mass (young, 61.8 ± 7.9 vs. older, 62.9 ± 10.9 kg; *p* = 0.83), and body surface area (BSA) (young, 1.7 ± 0.1 vs. older, 1.7 ± 0.2 m^2^; *p* = 0.80). There were significant differences in body fat percentage (young, 14 ± 3 vs. older, 19% ± 5%; *p* = 0.04) and predicted VO_2peak_ (young, 49.8 ± 8.7 vs. older, 33.3 ± 3.3 mL kg^-1^ min^-1^; *p* = 0.001). Baseline mean arterial pressure was higher in the older than in the young groups (young, 85 ± 7 vs. older, 94 ± 9 mmHg; *p* = 0.04).

All participants were recreationally active and free from illness and injury. Participants were non-smokers and had no history of cardiovascular disease, skin/sweat-related conditions, or neuromuscular disorders. All participants in the older age group were semiretired and worked two or three times per week (e.g., pruning, delivery, cleaning).

All experimental procedures were approved by the Human Research Ethics Committee of the National Institute of Occupational Safety and Health, Japan (2021N-1-8). The participants were informed of the experimental procedures and potential risks, and all signed consent forms. The study was conducted in accordance with the latest version of the *Declaration of Helsinki*, except for registration in a database.

### Experimental design

The volunteers visited the laboratory on four separate occasions which included a preexperimental/familiarization session and three experimental trials. The three trials (CON, DRY, and WET, for short, details in *Experimental protocol*) were randomized (at least 72 h between each one) and conducted between 10:00 to 15:00 h.

The volunteers refrained from consuming any beverages containing caffeine or alcohol from the night before the day of the experiment. They ate a light meal and drank ∼ 500 mL of water 2 h before the experiment and then reported to the laboratory. After voiding completely, each volunteer entered the thermoneutral room with an ambient temperature (T_a_) of 25°C and 50% RH.

### Preexperimental session

In this pre-session, all volunteers completed a submaximal exercise test on a treadmill (SportsArt Fitness T650, Woodinville, WA, USA) at a T_a_ of 25°C and 50% RH using a metabolic analyzer (AE-310 s, Minato Medical Science, Osaka, Japan) and electrocardiography (BSM-3400; Nihon Koden, Tokyo, Japan). The test was a modified ramp protocol consisting of a 2 min warmup of 2.5 km h^−1^ at 0% gradient followed by an increase in speed by 0.5 km/h every 2 min. After reaching 4.5 km h^-1^, the gradient was increased by 3% every 2 min until up to 15%. Then, the speed was increased by 0.5 km h^−1^ every 2 min at the 15% gradient. To ensure the safety of the older volunteers, the test was terminated when the subject’s steady-state HR reached 85% of the age-predicted maximum (220—age). The VO_2_ and HR data collected during the submaximal exercise test were extrapolated to estimate VO_2peak_ (American College of Sports Medicine, 2013). The same procedure was applied to the young volunteers to allow the comparison between the age groups.

To prescribe walking speeds and gradients for the main trials, the rate of metabolic energy expenditure (*M*) and heat production were estimated for each participant using data from the submaximal exercise test, as estimated by the following equation (Cramer et al., 2014):

$$M=\left({VO}_{2}\;\left(\left(e_{c}\;\left(\left(R\;–\;0.7\right)/0.3\right)\right)+\left(e_{f}\;\left(\left(1\;–\;R\right)/0.3\right)\right)\right)\right)\times 1000\;/\;60\;\left(W\right),$$

$$Heat\;production=\left(M-W\right)\;/\;BSA\;\left(W\;m^{-2}\right)$$

where *R* is the respiratory exchange ratio, *e*_*c*_ is energy equivalent of carbohydrate (21.13 kJ) per L of O_2_ consumed (L min^−1^), and *e*_*f*_ represents the energy equivalent of fat (19.69 kJ) per L of O_2_ consumed (L min^−1^). Heat production was estimated as the difference between *M* and the external work (*W*) and divided by BSA (W m^−2^). External work was calculated as follows (Gibson et al., 1979):

$$W=9.81\;v\times gradient\times mass\;\left(W\right)$$

where *v* is the selected treadmill speed (in m s^−1^), gradient is the treadmill gradient expressed as a decimal (e.g., 5% = 0.05), and mass is the body mass (in kilograms).

For each participant, measures of height in cm (YG-200P, Yagami, Nagoya, Japan), body mass in kg (F150S, Sartorius, Goettingen, Germany, Resolution 1 g), and body fat percentage *via* bioelectrical impedance (Body composition analyzer, InBody-270, Biospace, Seoul, Korea) were recorded.

### Experimental protocol

Participants wore a long-sleeved t-shirt (100% cotton, 160 g), a ventilation jacket (100% cotton, 430 g), undershorts (100% polyester, 80 g), work pants (100% cotton, 315 g), socks (100% cotton, 40 g), and shoes in all three trials. The intrinsic clothing insulation value of the jacket was 0.21 clo and that for the total clothing was 0.53 clo, was determined using a thermal manikin (Thermal manikin, Kyoto Electronics Manufacturing Co., Ltd., Tokyo, Japan). The size of the t-shirt was selected for each participant to adhere tightly to their body surface. To increase the wetted surface area, the long-sleeved type of t-shirt was selected. The ventilation jacket (KU91400, Kuchofuku, Tokyo, Japan) was long sleeved and equipped with two fans (8 cm blade diameter, 194 g) on the lower back (Supplementary Figure S1). The fans used a battery box (4 AA nickel-metal hydride, 140 g) and took in outside air. The airflow rate of the fan was 15.1 L s^−1^, which was set at the maximum of the apparatus because heat loss increases with ventilation flow rates (Yang et al., 2022). The jacket was tightened around the waist with an elastic band, and then, the air was exhausted from the cuff and neck openings. The airflow velocity underneath clothing was 4.5, 2.5, and 7.0 m s^−1^ at the cuff, in front of the neck, and behind the neck, respectively (measured using an anemometer; 6006, Kanomax Japan Inc., Suita, Japan).

After baseline measurements at 25°C (50% RH, <0.3 m/s air velocity), the t-shirt was soaked with 350 ± 5 mL of tap water (37°C) using an electric vaporizer in the WET trial, and water was never added later. This amount of water was chosen to saturate the t-shirt without leaving dry spots or dripping. To ensure the volume of water, the clothed body weight was measured before and immediately after the soaking (±5 g error) because the participants had trouble wearing a pre-determined and pre-soaked t-shirt. At the same time, the fans of the ventilation jacket were turned on in the DRY and WET trials. In the CON trial, the fans remained off. Immediately after each preparation of the clothing, the room temperature was elevated to 37°C (50% RH, <0.3 m/s air velocity) and stabilized within 10 min. The participants remained seated during the T_a_ transition, then they performed three 20-min bouts of walking exercise (Ex1, Ex2, and Ex3) separated by 10-min breaks (B1, B2, and B3). The walking was conducted at a predefined speed (all participants, 4.5 km h^−1^) and inclines (young, 6.6% ± 2.4%; older, 3.4% ± 2.1%) for a target heat production of 200 W m^-2^ on the treadmill (%VO_2_peak, young 39% ± 9%, older 54% ± 8%, as averaged across trials). During the breaks, drinking water (37°C) was provided *ad libitum*.

### Measurements

Rectal temperature (T_rec_) was measured continuously using a thermistor probe (701J, Nikkiso-Therm, Tokyo, Japan) self-inserted by the participant to 13 cm beyond the rectal sphincter. Skin temperature (T_skin_) at the chest, abdomen, back, forearm, upper arm, thigh, and lower leg were measured every 10 s using a temperature/humidity logger (DS1923 iButton Hydrochron, Maxim Integrated, San Jose, CA, USA) (MacRae et al., 2018; Kato et al., 2021). The temperature/humidity loggers were placed on the skin. The temperature sensor was located at the bottom the logger, measuring the skin surface temperature. The humidity sensor was located at the top of the logger, estimating the air humidity ca. 6 mm above the skin surface. The mean T_skin_ was calculated using the Ramanathan weighting system (Ramanathan, 1964). Clothing microclimate temperature and RH between the ventilation jacket and t-shirt around the chest were also measured using the two loggers placed on the inside of the jacket: one was set with the temperature sensor up and another was set with the humidity sensor up. To prevent touching the t-shirt, each sensor was covered with a 12-mm high plastic mesh cage. Sweat rate (SR) on the chest, back, forearm, and thigh were monitored by dew point hygrometry (OKS-04HM, ASE Giken, Nagoya, Japan). A ventilated plastic capsule was attached to each skin region. An index of skin blood flow on the chest, back, and forearm was measured using laser Doppler flowmetry (LDF; FLO-C1, Omegawave, Tokyo, Japan) located adjacent to the ventilated capsule at a sampling rate of 1/1,000 s. To avoid movements of the arms and trunk during the assessment of LDF, the participants held handrails for 30 s every 5 min. Because the large movements of the legs during walking disturb the LDF reading, measurements on the legs were not recorded. HR was recorded from an electrocardiogram (BSM-3400; Nihon Koden). Arterial blood pressures were measured using an electrosphygmomanometer (EBP-330, Minato Medical Science) every 5 min, with mean arterial pressure subsequently calculated as 1/3 (pulse pressure) + diastolic blood pressure. Cutaneous vascular conductance (CVC) was calculated as the LDF value divided by the mean arterial pressure and is expressed as a percentage change from the baseline value.

Metabolic rate was measured using the metabolic analyzer during exercise. Heat production during exercise was calculated using 5–20 min data in each walking period. Body mass was measured using a precision balance with ±1 g accuracy (F150S, Sartorius, Goettingen, Germany) before and after the experiment with the participant wearing only undershorts. Total sweat loss was calculated as follows: body mass loss + the weight of water ingested (kg). On completion of the experiment, participants removed the clothing immediately, and the weight of each clothing was measured. The moisture content of each clothing was calculated as the weight at the end of the experiment (g)—the weight of dry clothing before dressing (g).

Participants rated their thermal sensation, thermal comfort, thirst sensation, and fatigue separately by drawing a cross line on a visual analog scale (VAS). The subjective ratings of thermal sensation and comfort used a 20 cm VAS; “coldest” or “most uncomfortable” were scored as −10 cm, “hottest” or “most comfortable” were scored as 10 cm, and “neutral” was scored as 0 (Nakamura et al., 2008). For evaluating thirst and fatigue, the minimal VAS rating was scored as 0 (not thirsty or fatigued at all) and the maximal rating as 10 cm (extremely thirsty or fatigued). The ratings were conducted at baseline, immediately before T_a_ elevation and Ex1, and at the end of Ex1–Ex3 and B1–B3.

### Statistical analysis

An *a priori* sample-size calculation was performed (G*Power 3.1.9.6; Faul et al., 2007) using data from my previous investigations undertaken employing the similar experimental model (T_rec_, T_skin_, and Sweat loss). This indicated that we would need ≥8 participants per group to find statistical significance with an effect size of 0.4, a power of 0.9 and alpha set to 0.05.

Physical characteristics were compared between groups (i.e., young and older) using independent samples t tests (two-tailed). Single time point data (percentage of maximum HR, total sweat loss, the total volume of water ingested, body mass loss, and clothing mass) were evaluated by two-way analysis of variance (ANOVA) for repeated measurements with trials (CON, DRY, and WET) and groups (young and older). All other data were analyzed using a three-way repeated-measures ANOVA with trials, groups, and time. If a significant interaction was observed, *post hoc* paired-sample t tests were used to analyze further the effects of trials or groups by the Bonferroni method (physiological data) and Friedman’s test (perceptual data). Statistical significance was assumed at *p* < 0.05. Analyses were performed using commercially available software (IBM SPSS Statistics v. 21, IBM Corp., Armonk, NY, USA). All variables are reported as the mean ± standard deviation (SD).

## Results

The main effect of time was detected for T_rec_, mean T_skin_, regional SR, CVC, HR, heat production, the rating scores, and clothing microclimate temperature and RH in the young and older groups (all *p* < 0.05). The main effect of trial was significant for T_rec_, mean T_skin_, regional SR, CVC, HR, heat production, whole-body sweat loss, the total volume of water ingested, body mass loss, the rating scores of thermal sensation and comfort, the moisture content of t-shirts and other clothing, and clothing microclimate temperature and RH in both groups (all *p* < 0.05). A significant main effect of group was detected for mean T_skin_ and percentage of maximum HR (both *p* < 0.01). Furthermore, a significant interaction effect of time*trial (all *p* < 0.05) was detected for T_rec_, mean T_skin_, regional SR, CVC, HR, heat production, and the rating scores of thermal sensation and comfort, and clothing microclimate temperature and RH in both groups. The interaction effect of trial*group was detected for percentage of maximum HR, whole-body sweat loss, the total volume of water ingested, body mass loss, and the moisture content of t-shirts and other clothing (all *p* < 0.05). A time*trial*group interaction effect was detected only for mean T_skin_ (*p* < 0.05).

T_rec_ was lower in the DRY than in the CON trials at 110 min (Figures 1A, C, *p* < 0.05). T_rec_ in WET was lower than in both CON at 70–110 min (*p* < 0.05) and DRY at 50–110 min (*p* < 0.05). The mean T_skin_ was lower in WET than in CON and DRY throughout the experiments (Figures 1B, D, *p* < 0.01). The mean T_skin_ in DRY was lower than in CON from 40 to 110 min (*p* < 0.05). In WET, the mean T_skin_ was lower in the older than in the young groups from 40 to 110 min (*p* < 0.01). The mean T_skin_ responses were similar to T_skin_ on the chest, back, abdomen, upper arm, and forearm (Supplementary Figure S2). SR was lower in the WET than in the CON trials on the chest (Figures 2A, E, 40–110 min, *p* < 0.05), back (Figures 2B, F, 30–110 min, *p* < 0.05), forearm (Figures 2C, G, 35–110 min, *p* < 0.01), and thigh (Figures 2D, H, 75–110 min, *p* < 0.05). On the forearm, SR was lower in the WET than in the DRY trials (Figures 2C, G, 35–110 min, *p* < 0.05). Considering the changes in the CVC on the chest, back, and forearm (Supplementary Figure S3), before walking (5–20 min), changes in the CVC were lower in the WET than in the CON and DRY trials in all regions (all *p* < 0.01). During walking and breaks (25–110 min), changes in the CVC in all regions were lower in the WET than in the CON trials (all *p* < 0.01).

**FIGURE 1:**
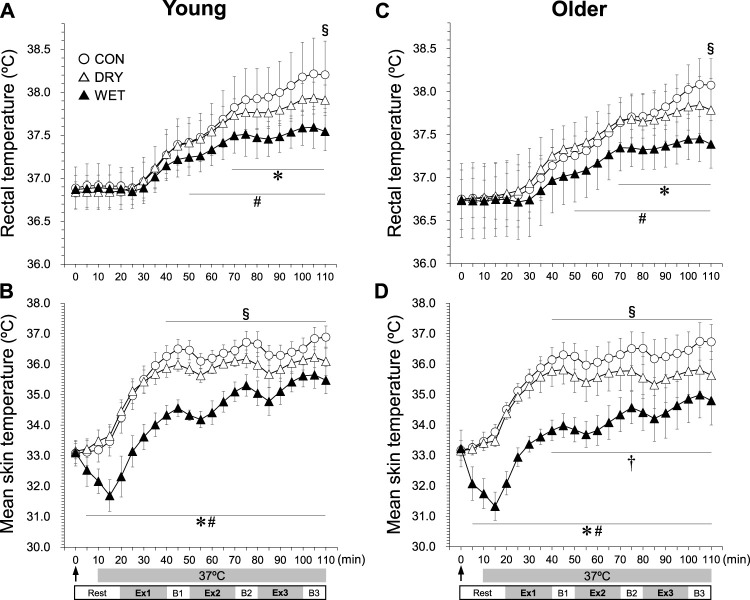
Rectal **(A,C)** and mean skin temperature **(B,D)** responses during three bouts of intermittent exercise (Ex1–3) in hot conditions (37°C, 50% RH) interspersed by three breaks (B1–3) in the young (left) and older (right) groups while wearing a dry inner t-shirt without ventilation garment fanning in the control (CON, open circles), a dry inner t-shirt with ventilation garment fanning (DRY, opened triangles), and a wetted inner t-shirt with ventilation garments fanning (WET, closed triangles). The arrow indicates the start of fanning (DRY) and wetting and fanning (WET). *Significantly different for CON vs. WET trial, *p* < 0.05. #Significantly different for DRY vs. WET trial, *p* < 0.05. §Significantly different for CON vs. DRY trial, *p* < 0.05. †Significantly different for young vs. older in the WET trial, *p* < 0.05. Data are expressed as the mean ± SD for eight participants.

**FIGURE 2:**
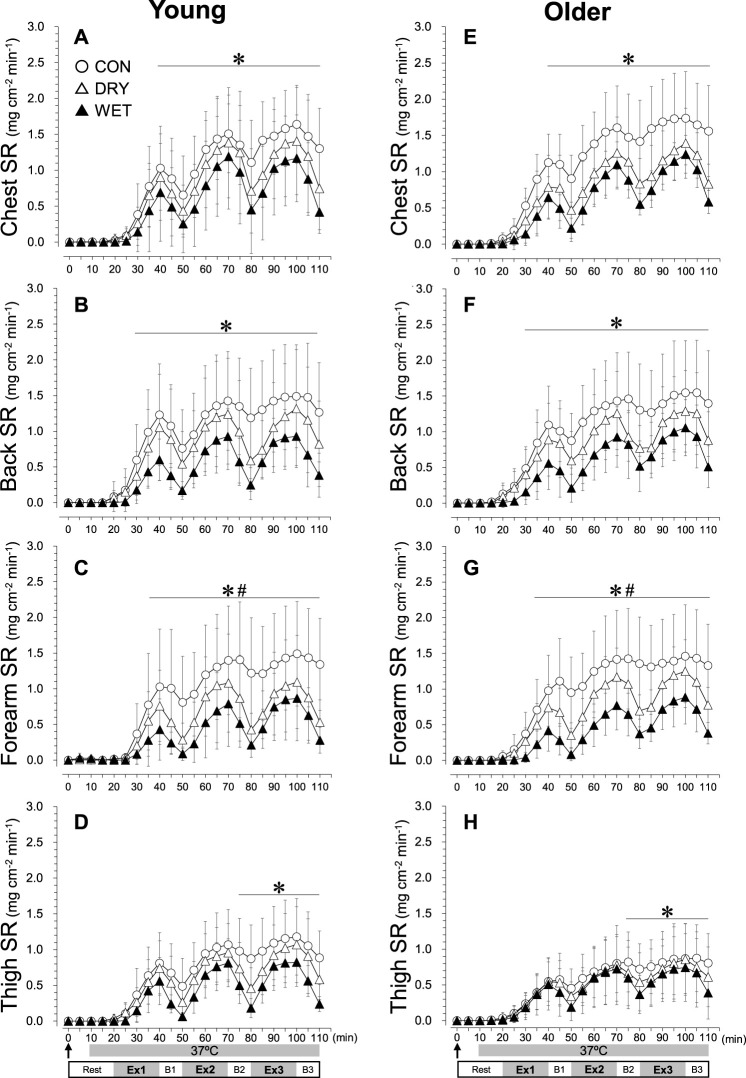
Regional sweating rate (SR) responses on the chest **(A, E)**, back **(B, F)**, forearm **(C, G)**, and thigh **(D, H)** during three bouts of intermittent exercise (Ex1–3) in hot conditions (37°C, 50% RH) interspersed by three breaks (B1–3) in the young (left) and older (right) groups while wearing a dry inner t-shirt without ventilation garment fanning in the control (CON, open circles), a dry inner t-shirt with ventilation garment fanning (DRY, opened triangles), and a wetted inner t-shirt with ventilation garment fanning (WET, closed triangles). The arrow indicates the start of fanning (DRY) and wetting and fanning (WET). *Significantly different for CON vs. WET trial, *p* < 0.05. #Significantly different for DRY vs. WET trial, *p* < 0.05. §Significantly different for CON vs. DRY trial, *p* < 0.05. Data are expressed as the mean ± SD for eight participants.

HR was lower in the DRY than in the CON trial (Figures 3A, B, 75–110 min, *p* < 0.05). In the WET trial, HR was lower than in the CON and DRY trials (30–110 min, both *p* < 0.05). The percentage of maximum HR during walking in each trial was higher in the older group (CON 85% ± 10%; DRY 79% ± 10%; WET 70% ± 10%) than in the young group (CON 73% ± 14%, DRY 67% ± 10%, WET, 60% ± 12%; all *p* < 0.05). In all trials, heat production in Ex2 and Ex3 was greater than in Ex1 (Figures 4A, B, all *p* < 0.05). In Ex3, heat production was lower in the WET trial than in both the CON and DRY trials (all *p* < 0.05).

**FIGURE 3:**
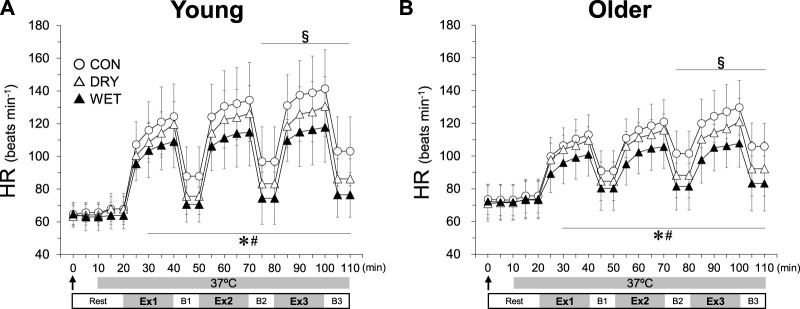
Heart rate (HR) responses during three bouts of intermittent exercise (Ex1–3) in hot conditions (37°C, 50% RH) interspersed by three breaks (B1–3) in the young **(A)** and older **(B)** groups while wearing a dry inner t-shirt without ventilation garments fanning in the control (CON, open circles), a dry inner t-shirt with ventilation garment fanning (DRY, opened triangles), and a wetted inner t-shirt with ventilation garment fanning (WET, closed triangles). The arrow indicates the start of fanning (DRY) and wetting and fanning (WET). *Significantly different for CON vs. WET trial, *p* < 0.05. #Significantly different for DRY vs. WET trial, *p* < 0.05. §Significantly different for CON vs. DRY trial, *p* < 0.05. Data are expressed as the mean ± SD for eight participants.

**FIGURE 4:**
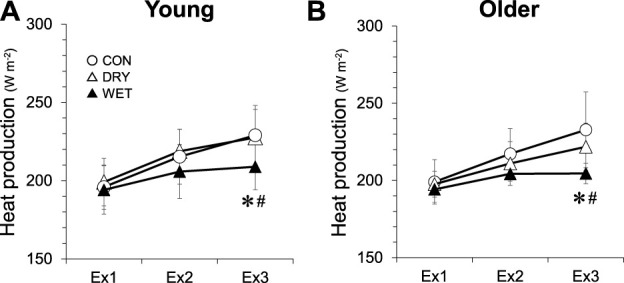
Heat production during three bouts of intermittent exercise (Ex1–3) in hot conditions (37°C, 50% RH) in the young **(A)** and older **(B)** groups while wearing a dry inner t-shirt without ventilation garments fanning in the control (CON, open circles), a dry inner t-shirt with ventilation garment fanning (DRY, opened triangles), and a wetted inner t-shirt with ventilation garment fanning (WET, closed triangles). *Significantly different for CON vs. WET trial, *p* < 0.05. #Significantly different for DRY vs. WET trial, *p* < 0.05. Data are expressed as the mean ± SD for eight participants.

Whole-body sweat loss in the CON trial was greater than in the DRY and WET trials, with values being greater in the DRY than in the WET trial in both groups (young: CON 1.03 ± 0.28 kg, DRY 0.80 ± 0.23 kg, WET 0.48 ± 0.22 kg; older: CON 1.04 ± 0.25 kg, DRY 0.85 ± 0.22 kg, WET 0.56 ± 0.14 kg; all *p* < 0.05). The total volume of water ingested during three break periods was greater in the CON than in the DRY and WET trials in the older group (CON 582 ± 217 mL, DRY 385 ± 163 mL, WET 366 ± 122 mL; both *p* < 0.05). No difference among the three trials was observed in the young group (CON 600 ± 308 mL, DRY 453 ± 160 mL, WET 445 ± 137 mL). In each break (B1–3), the volume did not differ among the three trials and between age groups (Supplementary Figure S4). Body mass loss was lower in the WET than in the CON and DRY trials in both groups (young: CON 0.42 ± 0.17 kg, DRY 0.35 ± 0.19 kg, WET 0.04 ± 0.11 kg; older: CON 0.46 ± 0.35 kg, DRY 0.47 ± 0.26 kg, WET 0.20 ± 0.22 kg; all *p* < 0.05).

**FIGURE 5:**
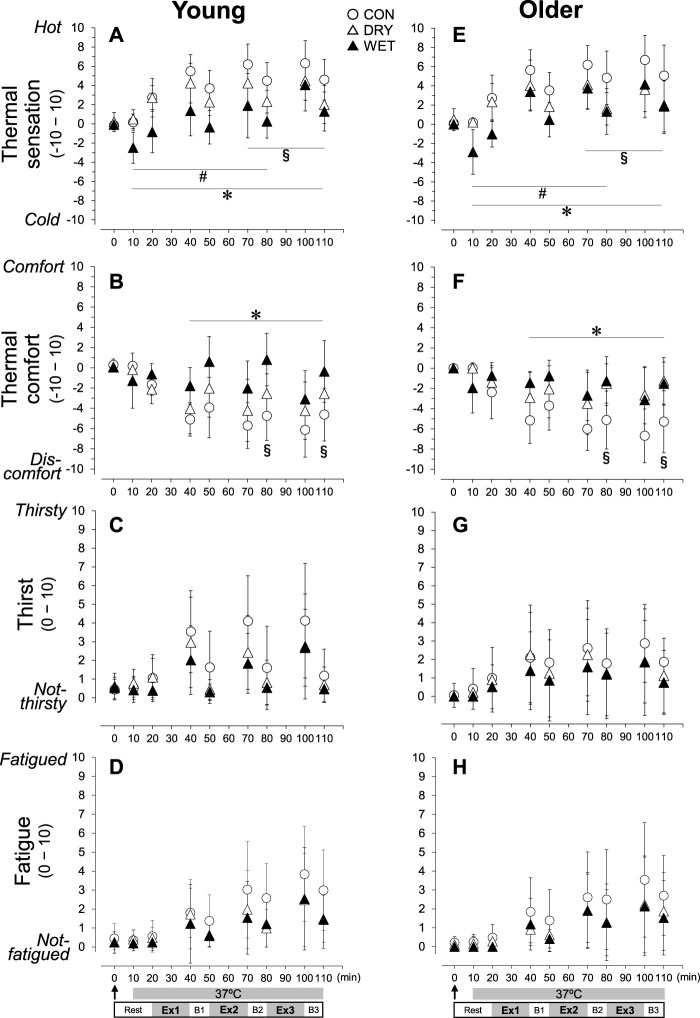
Perceptual responses of thermal sensation **(A, E)**, thermal comfort **(B, F)**, thirst **(C, G)**, and fatigue **(D, H)** during three bouts of intermittent exercise (Ex1–3) in hot conditions (37°C, 50% RH) interspersed by three breaks (B1–3) in the young (left) and older (right) groups while wearing a dry inner t-shirt without ventilation garment fanning in the control (CON, open circles), a dry inner t-shirt with ventilation garment fanning (DRY, opened triangles), and a wetted inner t-shirt with ventilation garments fanning (WET, closed triangles). The arrow indicates the start of fanning (DRY) and wetting and fanning (WET). *Significantly different for CON vs. WET trial, *p* < 0.05. #Significantly different for DRY vs. WET trial, *p* < 0.05. §Significantly different for CON vs. DRY trial, *p* < 0.05. Data are expressed as the mean ± SD for eight participants.

The thermal sensation rating in the WET trial decreased immediately after soaking the inner t-shirt (Figures 5A, E, *p* < 0.05). In the CON and DRY trials, the thermal sensation increased in Ex1–3 and B1–3 (all *p* < 0.05), but the rating scores in WET increased only in Ex3. The thermal sensation was lower in the DRY than in the CON from Ex2 to B3 (*p* < 0.05). The rating scores in WET was lower than in the CON throughout the experiment (*p* < 0.05) and DRY trials until Ex2 (*p* < 0.05). The thermal comfort rating decreased (i.e., discomfort increased) in Ex1–3 and B1–3 in the CON and DRY trials (Figures 5B, F; all *p* < 0.05), but only in Ex1–3 in the WET trial (all *p* < 0.05). The decreases in CON were greater than in DRY (B2 and B3, both *p* < 0.05) and WET (Ex1–3 and B1–3, all *p* < 0.05). The thirst sensation rating increased in Ex1–3 in all trials (Figures 5C, G; all *p* < 0.05). The fatigue rating increased in Ex1–3 and B1–3 in all trials (Figures 5D, H; all *p* < 0.05).

The moisture content of the t-shirts was greater in the CON than in the DRY and WET trials in the young group (CON 178 ± 64 g, DRY 44 ± 45 g, WET 58 ± 29 g; *p* < 0.05). In the older group, the moisture content of the t-shirts was greater in the CON than in the DRY and WET trials, with values being greater in the WET than in the DRY trials (CON 224 ± 88 g, DRY 83 ± 47 g, WET 111 ± 57 g; *p* < 0.05). The moisture content of other clothing (jacket, pants, undershorts, socks) was also greater in CON than in DRY and WET, with values being greater in WET than in DRY in the young group (CON 74 ± 40 g, DRY 19 ± 6 g, WET 18 ± 7 g; *p* < 0.05) and the older groups (CON 97 ± 41 g, DRY 29 ± 15 g, WET 42 ± 17 g; *p* < 0.05).

Clothing microclimate temperature increased immediately after room temperature was elevated to 37°C in all trials (Supplementary Figure S5). The increases in the DRY trial were greater than in the CON trial (*p* < 0.05), and the increases in the WET trial were lower than in both the CON and DRY trials (both *p* < 0.05). The RH of the clothing microclimate increased after the room temperature was elevated to 37°C in all trials (Supplementary Figure S5). The increases, which were greater than in the DRY and WET trials (all *p* < 0.05), were maintained throughout the experiment in the CON trials. The increases rapidly changed to decreases in the DRY trial but changed more slowly in the WET trial.

## Discussion

The present study evaluated whether wearing a water-soaked inner t-shirt with a fanned ventilation jacket would attenuate thermal and cardiovascular strains during exercise performed at moderate intensity work in hot and moderately humid conditions (37°C, 50% RH) in young and older individuals of work clothing worn. Consistent with our first hypothesis, wearing a water-soaked inner t-shirt while the ventilation jacket’s fans were on (WET) attenuated the increases in T_rec_, HR, and sweat loss during the 60-min intermittent exercise separated by three breaks in hot conditions compared with wearing a dry inner t-shirt with (DRY) or without (CON) ventilation jacket fanning in both age groups. Contrary to our second hypothesis, the mitigation of thermal and cardiovascular strains in WET did not differ between the young and older groups. These findings advance our understanding regarding effective and practical cooling strategies to reduce thermal and cardiovascular strains in hot occupations while wearing work clothing.

Differences in T_rec_ responses (Figures 1A, C) among the three trials likely reflected degrees of evaporative heat loss, while the precise heat loss values of a clothed person (Bröde et al., 2007; Havenith et al., 2008; Havenith et al., 2013) could not be calculated in the present study. Evaporative heat loss changes with a skin-air vapor pressure gradient, skin wettedness, the evaporative heat transfer coefficient, and air velocity (Gagge, 1937; Nelson et al., 1947). In the DRY conditions, natural sweating enhanced a certain degree of evaporative heat loss; attenuating thermal and cardiovascular strains by only using ventilation garments. This finding is in line with previous studies (Muza et al., 1988; Chen et al., 1997; Chinevere et al., 2008; Hadid et al., 2008; Barwood et al., 2009). In the WET trial, the prior moisture trapped in the inner t-shirt could supplement sweat production and lead to greater evaporative heat loss and mitigation of thermal and cardiovascular strains. Heat production in Ex3 was greater in CON and DRY relative to WET (Figure 4), although constant work intensity (walking speed and incline) for each volunteer was set at a target heat production (200 W m^−2^) that was determined in the preexperimental exercise test. This excess of heat production could be also related to the differences in T_rec_ responses. The water in the inner t-shirt in the WET trial dried gradually, the clothing microclimate temperature and RH reached the levels of the CON and DRY trials respectively in Ex3 (Supplementary Figure S5). Had the present study reapplied moisture to the inner t-shirt at the breaks, the benefits of wearing a water-soaked inner t-shirt while using ventilation garments could have been even greater. In practice, reapplying water is required to carry a worker through an entire day of labor.

Regional sweating was inhibited in the WET trial, largely on the chest, back, and forearm in both age groups (Figures 2A–C; Figures 2E–G). Thermoregulatory sweating is primarily controlled by T_core_ (Nadel, 1979), T_skin_ (Bullard et al., 1967), and skin blood flow (Wingo et al., 2010). All these factors were inhibited in the WET compared with both CON and DRY conditions throughout the experiment (Figure 1). Furthermore, because skin wettedness may affect the sweat gland response (Nadel and Stolwijk, 1973; Candas et al., 1979), the wetted inner t-shirt itself would reduce sweating on the chest, back, and forearm. Supplemental cooling by soaking an inner t-shirt before heat exertion can reduce natural sweat production on the torso and upper extremities covered by the ventilation jacket (∼55% coverage of the total body surface), which reduced total sweat loss in the WET trial. Workers in hot conditions often begin a working shift in a state of hypohydration and maintain a depleted hydration status throughout the day (Meade et al., 2015; Piil et al., 2018), although the American Conference of Governmental Industrial Hygienists (ACGIH) recommends frequent intake of a small amount of fluid, such as one cup (∼200 mL) every 20 min when working in warm environments (ACGIH, 2007). Here, the volunteers drank water *ad libitum* in the three break periods, while thirst sensations did not differ among the three trials in either group (Figures 5C, G). As a result, the ingested volume was not enough to balance the sweat lost in the CON and DRY trials in both groups (∼0.4 kg, total body mass loss including the ingested water). However, in the WET trial, body mass loss became nearly zero in the young group and was attenuated in the older group (∼0.2 kg). This mixed method of cooling (i.e., soaking an inner t-shirt and using ventilation garments) could be useful to prevent dehydration for workers in a hot environment. Because blood and urine samples could not be obtained in the present study, future studies should evaluate osmolarity and urine specific gravity.

Thermal sensation was less warm and thermal discomfort was lower in the WET than in the CON trial throughout both groups (Figures 4A, B, E, F). In the DRY trial, thermal perceptions were lower than in the CON trial after Ex2. Differences between the DRY and WET trial in thermal perceptions were observed from before exercise to Ex2. Thermal sensation is the information that pertains to external objects or the environment and is obtained only through warm or cold receptors in the skin; thermal comfort is affected by information from the skin and body core temperatures (Cabanac, 1979) and non-thermal factors (Havenith et al., 2002; Nagashima et al., 2018). Differences in T_rec_ and mean T_skin_ between the CON and WET trials were evident throughout (Figure 1). Increases in T_rec_ differed between the DRY and WET trials throughout (Figures 1A, C), but differences in mean T_skin_ between these trials diminished gradually toward the end of the experiment (Figures 1B, D). Although the rating scores of fatigue did not differ among the trials (Figures 4D, H), the relief of thermal sensation/discomfort by using ventilation garments might contribute to mitigation of workers’ psychological burden in hot conditions. Because the humidity sensation was not assessed in the present study, in the future, it should be evaluated how the soaking the inner t-shirt negatively affect humidity sensation in hot conditions.

Contrary to the hypothesis, thermal and cardiovascular responses in each trial were not different between the young and older groups, though mean T_skin_ responses in the WET trial were lower in the older than in the young group (Figures 1B, D). The mitigation effects of the mixed cooling method were similar between the two age groups. In the literature, it is unclear whether the effects of electric fan use on thermal strain differ between young and older individuals. Gagnon et al. (Gagnon et al., 2016; Gagnon et al., 2017) demonstrated that electric fan use in older individuals resulted in greater thermal and cardiovascular strains compared with young individuals during resting exposure to extreme heat and humidity (42°C, 30%–70% RH) while wearing only shorts. They presumed that the age-related differences were associated with a reduced sweating capacity in older adults (Gagnon et al., 2016; Gagnon et al., 2017). On the other hand, Wright Beatty et al. (2015) showed that mitigations in T_rec_ and T_skin_ responses by increasing airflow during exercise in hot conditions (35°C, 60% RH) were similar between young and older adults while wearing standard work clothing. They observed that sweating responses during exercise did not differ between the young and older groups, although heart rate and cutaneous vascular responses were attenuated by increasing airflow in the young compared to older groups (Wright Beatty et al., 2015). The discrepancy between the two studies might relate to a reduced level of sweating capacity in older adults. Here, sweat loss and regional SR were not different between the young and older groups in each trial. If sweating responses were attenuated in the older groups more than in the young group, the effects of ventilation garments use only (i.e., the DRY trial) would be smaller in the older groups. However, supplemental sweating by soaking an inner t-shirt compensated the age-related decline in sweating, thus the mixed-method cooling (i.e., the WET trial) could not lead to differences in effectiveness between the two age groups.

### Considerations

These findings are limited to the environmental conditions tested. If the air temperature surpasses T_skin_ to a great extent, ventilation garment fanning would enhance convective heat gain and exceed evaporative heat loss. In fact, while wearing a wetted t-shirt, facing an electric fan at 42°C (34% RH) caused greater T_core_ increases than no fanning (Cramer et al., 2020). Foster et al. (2022) showed the critical environmental limits for electric fan use during work in hot conditions. In their models, RH is also an important factor and not only air temperature for occupational heat strain. When the air temperature is ≥35°C, fans are ineffective and potentially harmful when RH is below 50% (∼2.8 kPa), i.e., in dry-heat climatic conditions. A high wind would be beneficial against thermal strain for humid-heat climatic conditions (RH ≥ 50%). Furthermore, the critical environmental limits were modified by SR; beneficial environmental zones broadened in elevated SR (1,500 g h^−1^) and were narrower in cases of depressed SR (500 g h^−1^) (Foster et al., 2022). Evaluating the critical environmental limits of using ventilation garments with a wetted inner t-shirt represents an important issue for future research because cooling efficiency may differ between an electric fan and ventilation clothing uses.

Second, the present study selected for the control condition of wearing the ventilation jacket without fanning as standard work clothing. Although the intrinsic clothing insulation of the jacket was relatively low (0.21 clo, single-layered 100% cotton), wearing it can restrict heat dissipation from the trunk and arms in hot conditions. Because there are work situations where light clothing is permitted for heat safety, a trial without the jacket (i.e., wearing only a t-shirt) should have been evaluated.

Third, the older participants were healthy with no history of cardiovascular disease and were not taking any medications at the time of participation. Notley et al. (2021) reported that type 2 diabetes and hypertension in older adults attenuated the tolerance to prolonged work in hot conditions, whereas no differences in thermoregulatory responses were observed compared with healthy older adults. Future studies should explore the cooling strategies during work in heat for older adults with common chronic disease. In addition, because sex, fitness level, hydration and heat acclimatization state influence thermoregulatory function in hot conditions (Ioannou et al., 2022), future studies should evaluate whether cooling interventions for individuals with each factor are effective in reducing thermal strain.

Finally, the present findings are restricted to laboratory settings that do not include sun exposure, natural wind flow, and job task requirements and complexities. Furthermore, some industrial sectors need more severe work intensities (higher metabolic demand) and extended work shifts. Future research should consider investigating whether strategies to supplement sweat production and ventilation garments can effectively mitigate thermal strain in real occupational fields of observational and intervention studies (Ioannou et al., 2021).

## Conclusion

For both young and older individuals during moderate-intensity work in hot conditions while wearing work clothing, the combination of wearing a water-soaked inner t-shirt and ventilation garments mitigated the rise in core temperature and heart rate and reduced skin temperature and whole-body sweat losses.

## Data Availability

The original contributions presented in the study are included in the article/Supplementary Material, further inquiries can be directed to the corresponding author.
