# Supplementary figures and images for: Effects of wetted inner clothing on thermal strain in young and older males while wearing ventilation garments
Source: Front Physiol. 2023 Feb 22;14:1122504. doi: 10.3389/fphys.2023.1122504 (PMC9992724; doi:10.3389/fphys.2023.1122504)

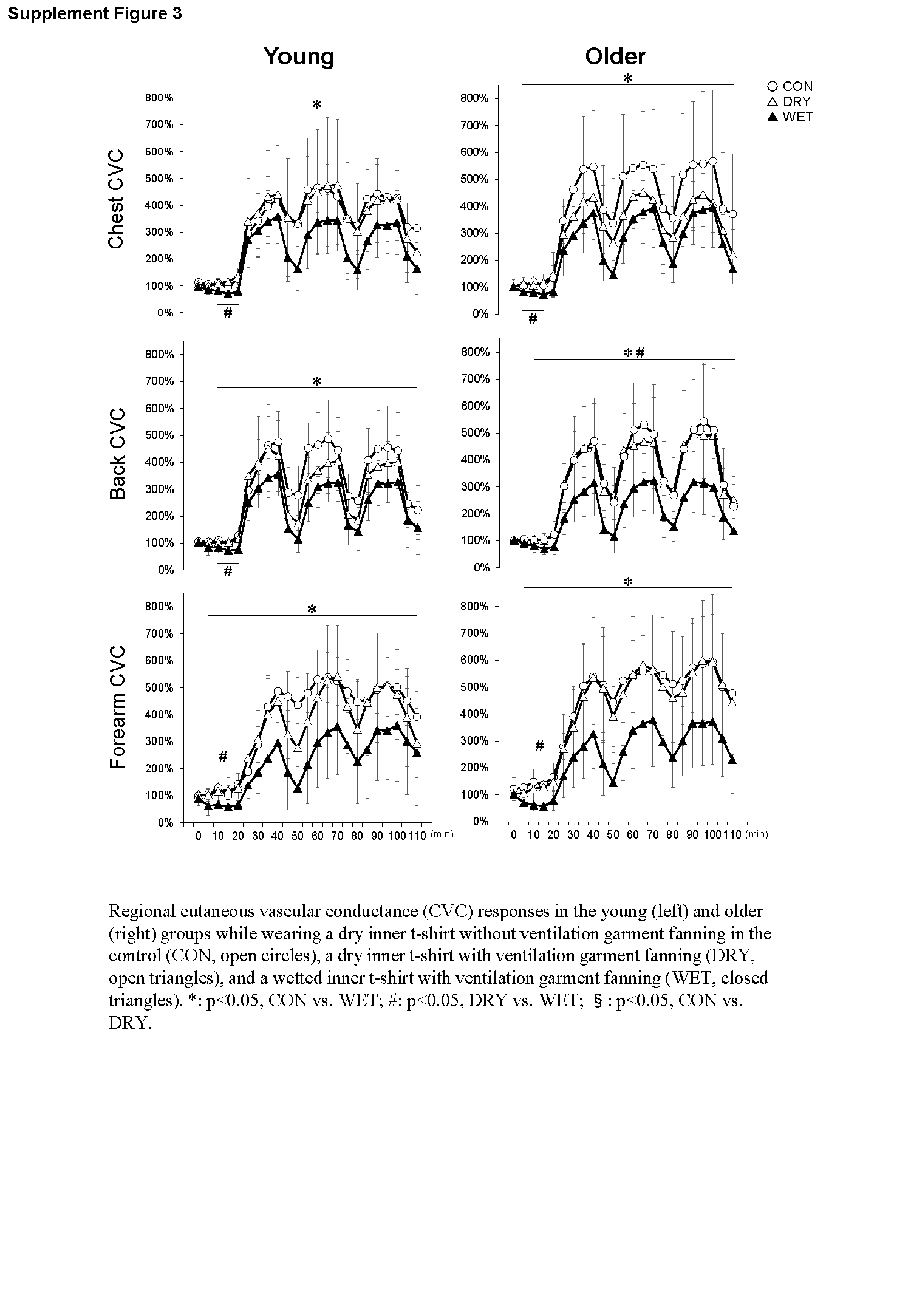

Supplement: Supplementary file 1 [file Image3.TIFF]

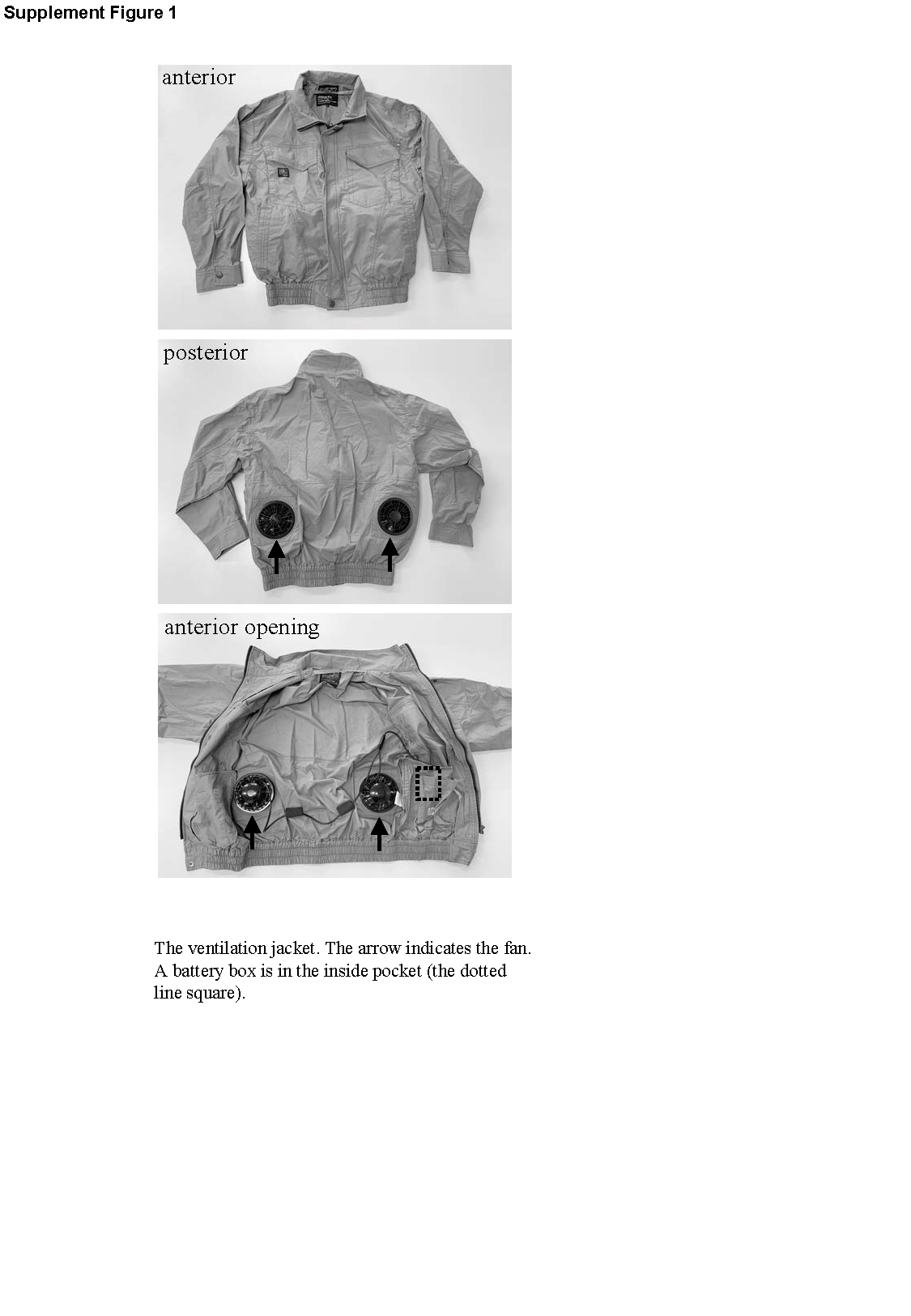

Supplement: Supplementary file 2 [file Image1.TIFF]

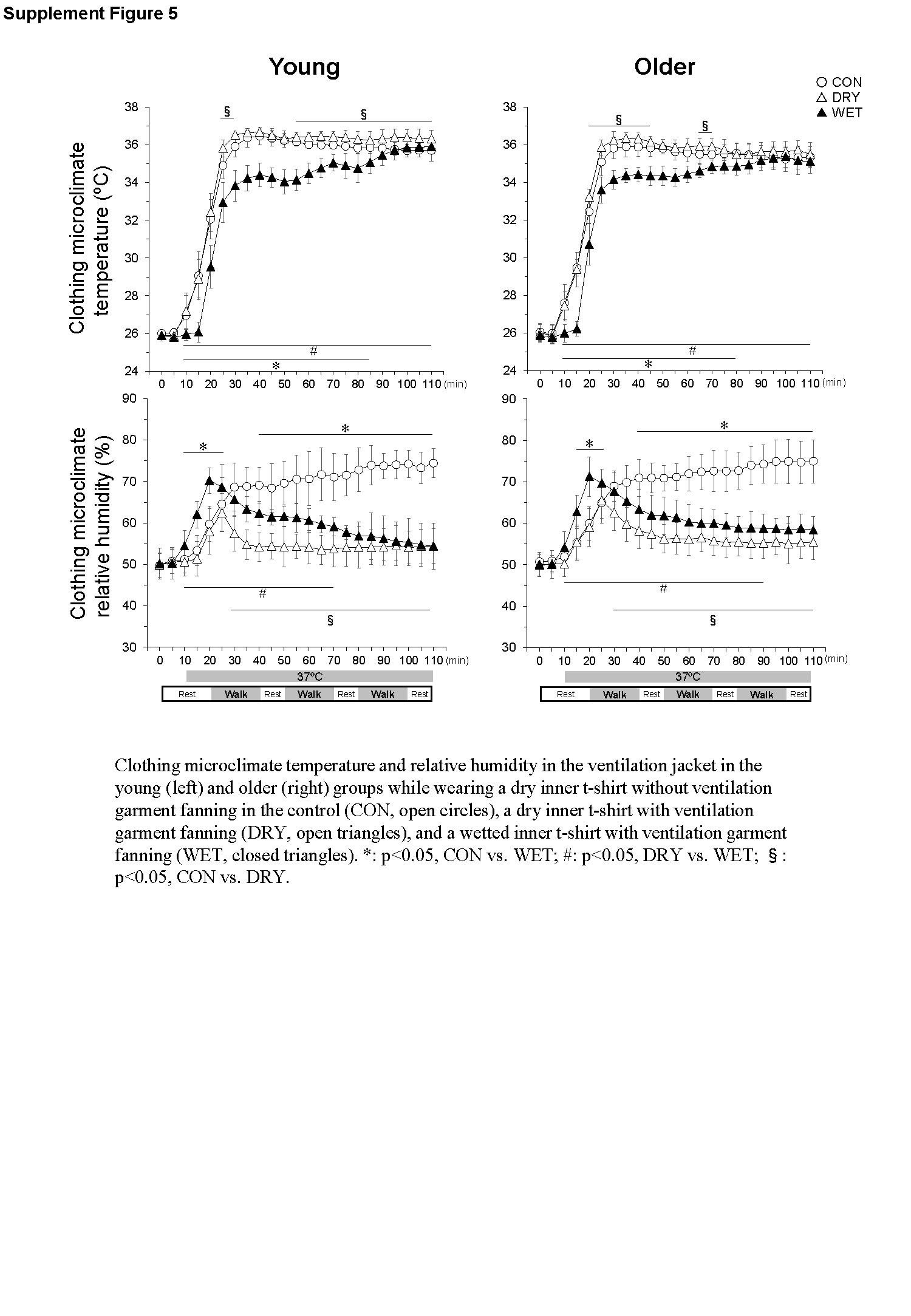

Supplement: Supplementary file 3 [file Image5.TIFF]

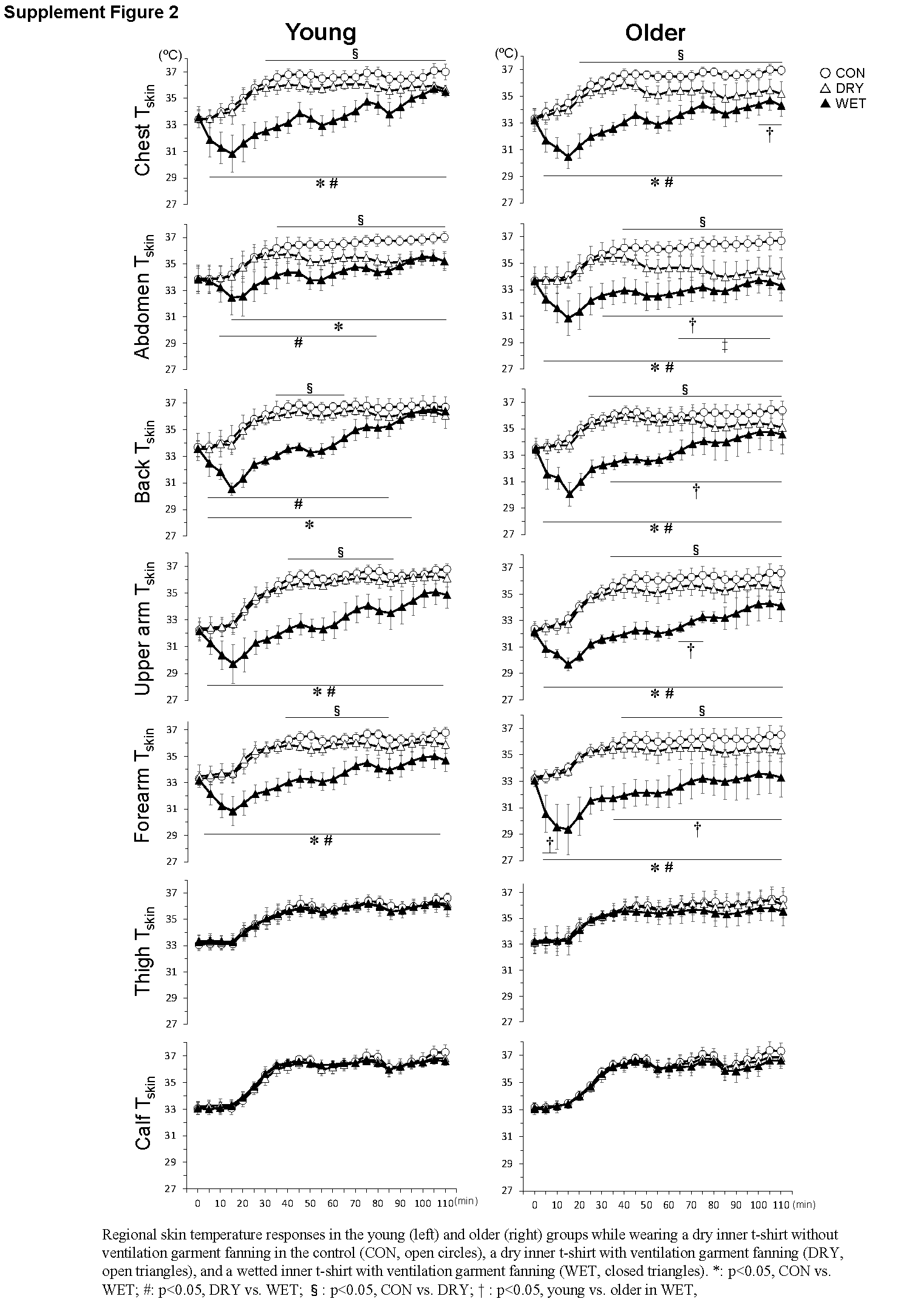

Supplement: Supplementary file 4 [file Image2.TIFF]

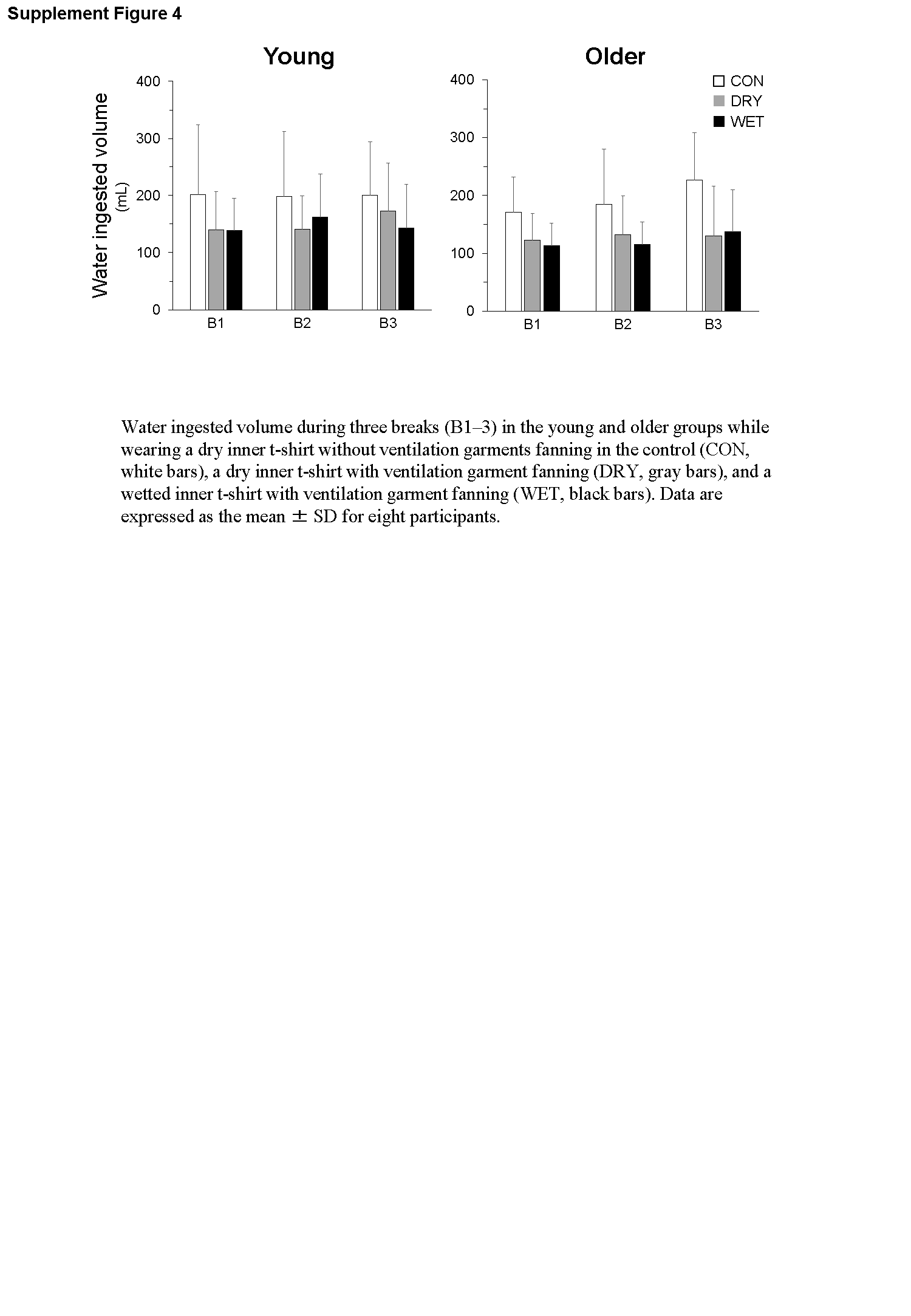

Supplement: Supplementary file 5 [file Image4.TIFF]
